# A word of caution: bilateral axillofemoral bypass could not provide sufficient blood flow in a patient who underwent aortic resection for aortoesophageal fistula: a case report

**DOI:** 10.1093/jscr/rjab356

**Published:** 2021-10-25

**Authors:** Ryo Okubo, Tomonori Shirasaka, Keisuke Shibagaki, Hiroyuki Kamiya

**Affiliations:** Department of Cardiac Surgery, Asahikawa Medical University, Asahikawa, Japan; Department of Cardiac Surgery, Asahikawa Medical University, Asahikawa, Japan; Department of Cardiac Surgery, Asahikawa Medical University, Asahikawa, Japan; Department of Cardiac Surgery, Asahikawa Medical University, Asahikawa, Japan

## Abstract

An 81-year-old man was transferred to our hospital for a ruptured infected descending aortic aneurysm. An emergency thoracic endovascular aortic repair was performed, but a computed tomography scan 7 days later revealed an aortoesophageal fistula. The establishment of extracorporeal circulation using the femoral artery and utilization of the omentum was considered difficult. We performed bilateral axillofemoral bypass followed by descending aortic resection and esophagectomy. However, the patient’s circulatory insufficiency worsened, and he died on the 18th postoperative day.

In the treatment of aortoesophageal fistula, bilateral axillofemoral bypass is not recommended as an alternative to descending aortic replacement.

## INTRODUCTION

Aortoesophageal fistula is a critical condition. Recently, there have been several promising reports on its treatment, including resection of the aneurysm and esophagus, *in-situ* reconstruction of the descending aorta and omental flap installation [1, 2]. However, such radical surgery using cardiopulmonary bypass is quite invasive, and there would be some patients who cannot tolerate this procedure.

We performed bilateral axillofemoral bypass and radical resection of the infected aneurysm and the esophagus without extracorporeal circulation in a patient with aortoesophageal fistula. The patient died of multi-organ failure due to insufficient blood flow to the lower body. Here, we present our experience as a word of caution.

## CASE REPORT

**Figure 1:**
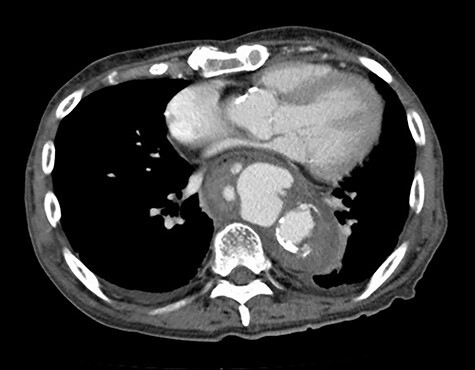
CT at the time of admission shows irregular shape of the aneurysm.

The patient was an 81-year-old man with a history of maintenance dialysis due to diabetic nephropathy, right hemicolectomy due to transverse colon cancer, coronary artery bypass grafting and abdominal aorta-femoral-popliteal bypass surgery due to peripheral arterial disease. The patient complained of anorexia for the last 1 month, and a screening computed tomography (CT) scan revealed a ruptured descending aorta (Fig. 1). He was transferred to our hospital for surgical management. At the time of admission, his vital signs were stable, but blood tests indicated a systemic infection, given that his leukocyte count was 14 510/μl and C-reactive protein was 27.92 mg/dl.

After admission, an emergency thoracic endovascular aortic repair (TEVAR) was performed using 34 × 150 mm and 31 × 150-mm sized endografts (CTAG, W. L. Gore & Associates, Flagstaff, AZ, USA). Based on the irregular shape of the aneurysm and available laboratory data, an infectious aortic aneurysm was suspected, and antimicrobial agents were administered since the time of admission.

**Figure 2:**
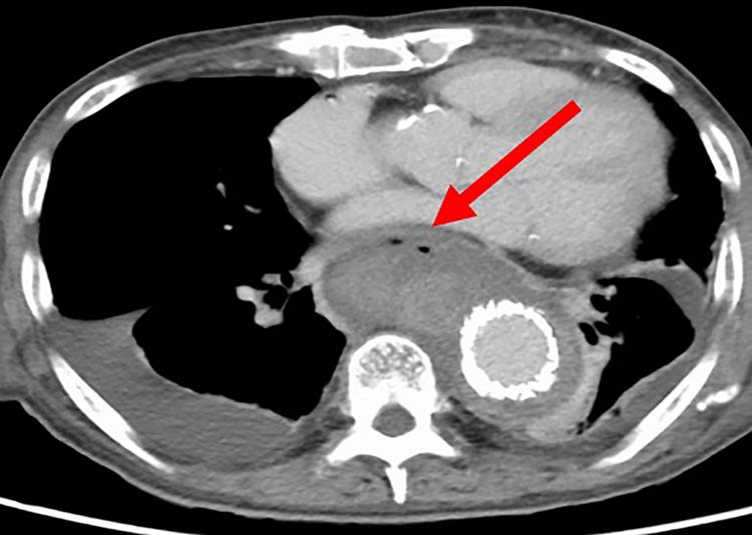
CT seven days after TEVAR. Arrow shows gas images inside the aortic aneurysm.

**Figure 3:**
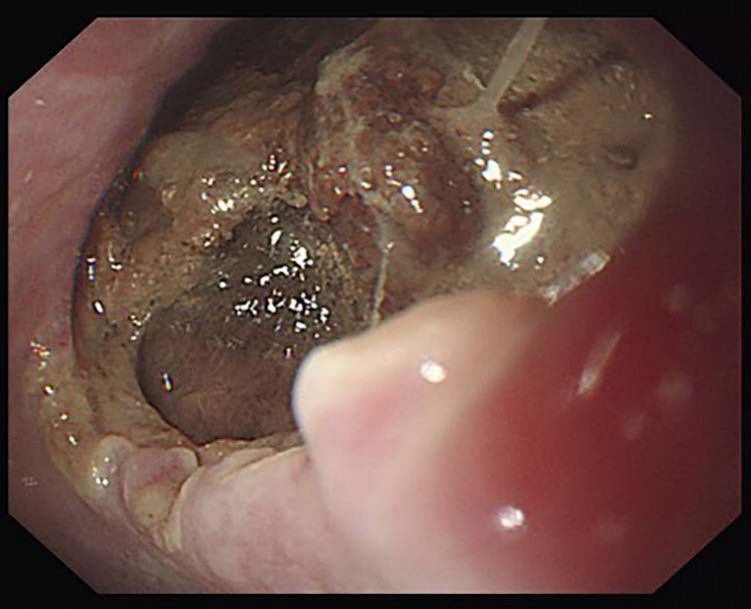
Esophagoscopy revealing an aortoesophageal fistula covered by necrotic tissue.

Seven days after TEVAR, the patient developed fever, and a CT scan showed bilateral iliopsoas abscesses and gas images inside the aneurysm (Fig. 2). We decided to perform upper gastrointestinal endoscopy and CT-guided drainage for abscesses. Endoscopy showed perforation of the esophageal wall and accumulation of necrotic tissue (Fig. 3).

The establishment of extracorporeal circulation using the femoral artery as the blood delivery route was considered difficult due to peripheral artery disease, and it was deemed impossible to use omental flap due to a history of right hemicolectomy. Therefore, *in-situ* reconstruction of the descending aorta seemed to be impossible, and we decided to perform bilateral axillofemoral bypass followed by descending aortic resection and esophagectomy.

The surgery was performed under general anesthesia. First, bilateral axillofemoral bypass surgery was performed through the subcutaneous route using an 8-mm polytetrafluoroethylene graft (PROPATEN, W. L. Gore & Associates, Flagstaff, AZ, USA) that was comparable with the diameter of the subclavian arteries.

Subsequently, the patient was placed in the right lateral supine position and the descending aorta was exposed by opening the left posterolateral side of the chest. The aorta was resected, and the ends were closed by suturing. The proximal side was reinforced with a bovine pericardial patch. The remaining descending aorta was incised longitudinally, and the stent graft was removed. The gastrointestinal surgeon then performed an esophagectomy. Because of continuous oozing of blood, the chest was temporarily closed with gauze packing. Finally, a cervical esophagostomy and gastrostomy were performed. The total operative time was 14 h and 14 min, and the blood loss was 5853 ml. Pathology was suggestive of an infectious aortic aneurysm, and blood cultures repeatedly showed *Staphylococcus aureus*. On postoperative Day 4, the chest was closed.

The patient’s consciousness gradually improved; however, we suspected prolonged ventilator management and performed a tracheostomy on the 13th day after surgery. However, his general condition gradually worsened, resulting in multi-organ failure. The patient developed critical ischemia of the extremities. The patient’s circulatory insufficiency worsened, and he died on the 18th postoperative day.

**Figure 4:**
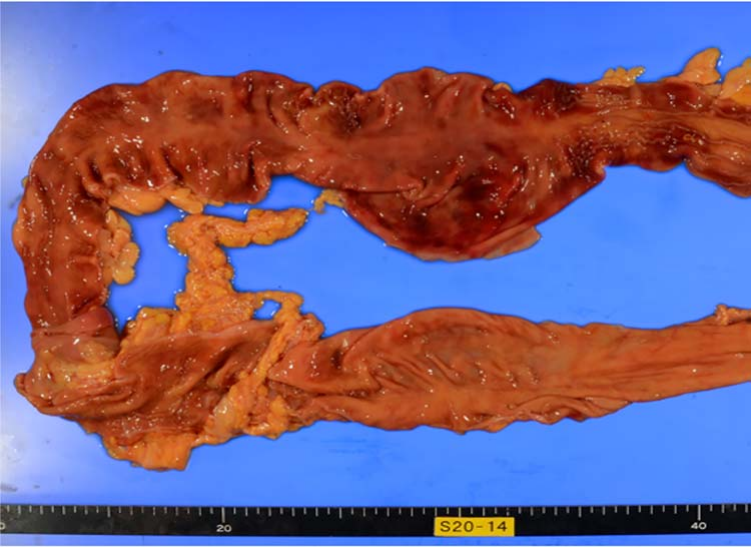
Photo of pathological autopsy. The colon shows ischemic changes such as hemorrhage, necrosis of the mucosa and loss of wrinkles.

The pathological autopsy showed black discoloration of the extremities and intestinal ischemia (Fig. 4), and the cause of death was diagnosed as multiple organ failure due to circulatory failure.

## DISCUSSION

Aortoesophageal fistula is a fatal disease. In cases of instability, emergency TEVAR may be considered. However, TEVAR can only be a temporary solution, and early radical surgery should be considered. Yamazato *et al.* and other researchers have reported good results with descending aortic replacement including omental flap installation using partial extracorporeal circulation as a radical treatment for aortoesophageal fistula [1, 2]. However, for cases in which omental flap installation is difficult, as in the present case, there is no valid treatment strategy, especially with respect to techniques preceding non-anatomical bypass.

There have only been a few case reports in which axillofemoral bypass has been performed due to difficulty replacing the descending aorta. Hageman *et al.* described a case of a descending aortic replacement for an aortic-esophageal fistula in a patient who developed a graft infection. In this case, unilateral axillofemoral and femorofemoral bypass were performed, followed by descending aortic resection. However, the patient developed postoperative refractory hypertension due to massive afterload and died of acute type A aortic dissection [3]. In the present case, we performed bilateral axillofemoral bypass, and the patient did not encounter the problem of increased afterload.

On the other hand, Stallone *et al.* reported two patients with aortic aneurysm treated with axillofemoral bypass and resection of the descending aorta. One patient died of cardiac failure, but the other survived without complications [4]. Their report implies that axillofemoral bypass and resection of the descending aorta could be an alternative option. In patients with aortoesophageal fistula, on-going infection and demand for blood supply to the lower body is higher than in non-infected patients, which may explain why axillofemoral bypass plus resection of the descending aorta failed in the present case.

Other methods such as ascending aorta-abdominal aortic bypass have been reported in cases with good outcomes [5]. In the present case, the patient underwent coronary artery bypass grafting previously, and this option was thus impossible.

In the treatment of aortoesophageal fistula, bilateral axillofemoral bypass as an alternative to descending aortic replacement is not recommended, especially in patients with advanced peripheral artery disease, because it cannot ensure adequate blood flow.
